# Polymorphisms in immunosuppression-related genes are associated with AML

**DOI:** 10.3389/fimmu.2025.1530510

**Published:** 2025-02-05

**Authors:** Mingying Li, Jingjing Ye, Mengyuan Chang, Lei Feng, Tingting Liu, Di Zhang, Yuyan Wu, Yuechan Ma, Guangqiang Meng, Chunyan Ji, Tao Sun

**Affiliations:** ^1^ Department of Hematology, Qilu Hospital of Shandong University, Cheeloo College of Medicine, Shandong University, Jinan, Shandong, China; ^2^ Shandong Key Laboratory of Hematological Diseases and Immune Microenvironment, Qilu Hospital of Shandong University, Jinan, Shandong, China

**Keywords:** AML, immunosuppression-related genes, treatment response, prognosis, SNPs

## Abstract

**Background:**

Acute myeloid leukemia (AML) is a hematologic malignancy with poor overall survival (OS). The immunosuppressive microenvironment significantly impacts AML development and chemoresistance. Despite new immunotherapeutic strategies entering standard clinical care for various tumors, progress in AML remains poor. Multi-omics analyses, such as single-cell transcriptomics, have revealed many potential new targets to improve AML prognosis from an immunological perspective.

**Methods:**

DNA from 307 AML patients and 316 healthy individuals were extracted. We detected nine single nucleotide polymorphisms (SNPs) in five immunosuppression-related genes (CIITA, CD200, CD163, MRC1 and LILRB4) in these samples. SNP genotyping was performed on the MassARRAY platform. We then analyzed the relationship between these SNPs and AML susceptibility, treatment response, and prognosis.

**Results:**

Our findings indicated that rs4883263 in the CD163 gene is a protective factor for AML susceptibility and chromosomal karyotype abnormalities. Additionally, rs4883263 in CD163 was related to low PLT count at diagnosis, while rs2272022 in CD200 was protective against low PLT count. rs4780335 in CIITA was associated with high WBC count at diagnosis and worse OS. Furthermore, rs1048801 in LILRB4 was linked to worse AML treatment response, lower OS, and may be an independent prognostic risk factor for AML. Lastly, expressions of CD163, CIITA, LILRB4, and CD200 were higher in AML patients than that in normal controls.

**Conclusions:**

Our findings on SNP associations in AML immunosuppression-related genes provide important reference points for predicting treatment outcomes in AML patients.

## Introduction

Acute myeloid leukemia (AML) is a hematologic malignancy characterized by poor overall survival (OS). It has become increasingly clear that AML development and progression are intricately linked to dysregulated immune responses. Notably, recent studies have illuminated how leukemic cells reshape and manipulate the tumor microenvironment, establishing a specialized niche that facilitates their survival and confers resistance to therapies. AML cell escape strategies involve direct adaptation of AML cells to evade immune recognition and tumor cell-mediated alterations in the immune cell lineage, including T cells, natural killer (NK) cells, and dendritic cells (DCs) (1–3).

The graft-versus-leukemia effect happened during allogeneic hematopoietic stem cell transplantation (HSCT) for AML treatment, wherein donor-derived immune cells eradicate leukemic cells, marked a paradigm shift in cancer therapy and has since advanced into targeted immunotherapeutic approaches, including chimeric antigen receptor (CAR) T cells, CAR-NK cells, bispecific T-cell engagers (BiTEs), and immune checkpoint inhibitors (4). Different immunotherapeutic concepts are under evaluation in AML clinical trials, but reported results show modest impact on the disease (5–7). Regarding immune checkpoint inhibitors, although sabatolimab targeting TIM3 showed good safety and tolerability and some durable clinical efficacy in a preliminary phase I/b study in combination with HMA (8), a recent study using the anti-PDL1 antibody durvalumab in elderly patients failed to demonstrate any additional clinical effect over azacitidine (9). This suggests that predicting responders remains difficult and that new biomarkers need to be established to predict clinical outcomes.

With the advent of multi-omics analyses, such as single-cell transcriptomics (scRNA-Seq), it is possible to better decipher the AML immunologic microenvironment and to envision more tailored immunotherapeutic strategies for the future of AML treatment. Several articles report varying degrees of T-cell dysfunction in BM samples from AML patients due to suppressive molecules expressed by AML cells using multi-omics analysis, which significantly affect the treatment response and prognosis of AML. For example, suppression of CD4+ T-cell activation by AML cells at onset is strongly associated with unfavorable outcomes in AML patients receiving standard chemotherapy, closely related to the CIITA (the master regulator of MHC class II expression), CD200 and MRC1 (Macrophage mannose receptor 1) expression in AML cells (10). Additionally, monocyte-like AML cells effectively suppress T-cell activation by expressing a series of immunomodulatory genes, such as antigen-presenting components MRC1 and CD163, leading to altered T-cell phenotype and shaping an immunosuppressive AML microenvironment (11). As in monocyte AML cells, LILRB4 (leukocyte immunoglobulin-like receptor B4), a marker for mononuclear leukemia, coordinates the tumor invasion pathway by mediating T cell suppression (12). All these molecules may become new potential targets to improve the prognosis of AML patients from an immunological perspective.

AML is a genetically complex, dynamic disease (13). Identifying genetic variants and analyzing their effects may help us to better understand their impact on gene function and disease development. For AML, gene mutations such as DNMT3A, TET2, and ASXL1 recommended by the European Leukemia Network are common in clonal hematopoiesis and appear to be relatively early events in the development of leukemia (14), which also reveals the great potential of SNPs in the diagnosis, treatment, and prognosis assessment of AML. Currently, the roles of immune-related SNPs have been investigated in AML patients and susceptibility, prognosis and survival-related SNPs has been identified (15–18). To better understand the unique etiology and treatment efficacy heterogeneity of AML, we investigated the contribution of AML immunosuppression-related SNPs. We analyzed the relationship between disease susceptibility, baseline data, treatment response, survival, risk stratification and these SNPs with the aim of helping to guide AML stratification and treatment.

## Methods

### Study subjects

Genomic DNA was isolated from bone marrow mononuclear cells (BMMCs) by the standard salting-out method using the standard salting-out method with the TIANamp Blood DNA kit (Tiangen Biotech, Beijing, China). SNP genotyping was performed using the Sequenom iPLEX and MALDI-TOF-based MassARRAY platform (BGI Tech, Beijing, China), which employs multiplex PCR, locus-specific single-base extension, and MALDI-TOF spectrometry, allowing analysis of up to 30 SNPs in a single reaction well. Primers were designed using Assay Design Suite version 2.0 (Agena Bioscience, San Diego, CA, USA), available from the manufacturer’s online tools (https://www.mysequenom.com/Tools). Six negative and six positive controls were included with the study samples to ensure accuracy. Moreover, 16 samples selected from the study group were detected in two independent test panels, achieving 99% reproducibility.

### Clinical end point definitions

The clinical endpoints used in this evaluation were defined as follows (1): Complete remission (CR), trilineage hematopoietic recovery with <5% blasts in the marrow after induction 2 (2); Relapse, after CR, peripheral blood leukemia cells or bone marrow original cells ≥5% (excluding other causes like bone marrow regeneration post-consolidation chemotherapy) or extramedullary leukemia cell infiltration (3); OS, time from enrollment to death, with living patients reviewed at the date of the last follow-up.

### Characteristics of the study group

For detecting genetic polymorphisms, 307 AML patients (166 males and 141 females) with a median age of 48 (13–87) years and 316 healthy controls (117 males and 199 females) with a median age of 40 (20–88) years were recruited into the study. Final AML diagnoses and classification were confirmed using the French-American-British (FAB) classification. AML patients were grouped into favorable, intermediate, or adverse prognosis categories based on karyotypic and molecular abnormalities according to National Comprehensive Cancer Network (NCCN) guidelines. Initial diagnosis were made from October 6, 2010 to December 15, 2021. Control subjects, recruited concurrently with case subjects, were randomly selected from hospital volunteers and matched by age and gender distribution. The study was approved by the Medical Ethics Committee of Qilu Hospital of Shandong University. All participants provided written informed consent before enrollment, in accordance with the Declaration of Helsinki. The characteristics of AML patients and healthy controls are shown in **Table 1**, along with bone marrow blast, routine blood counts, risk stratification, and treatment response data.

**Table 1:** Demographic and clinical characteristics.

Characteristic	Case n (%)	Control n (%)
**Age (years, median, range)**	48 (13–87)	40 (20–88)
<60	237 (77)	294 (93)
≥60	70 (23)	22 (7)
Gender		
Male	166 (54)	117 (37)
Female	141 (46)	199 (63)
FAB classification		
M0	2 (0.7)	n.a.
M1	5 (1.6)	n.a.
M2	20 (6.5)	n.a.
M3	52 (16.9)	n.a.
M4	51 (16.6)	n.a.
M5	171 (55.7)	n.a.
M6	2 (0.7)	n.a.
M7	0	n.a.
Unknown	4 (1.3)	n.a.
Bone marrow blast		
**Median (%)**	83	n.a.
<70%	93 (30)	n.a.
≥70%	214 (70)	n.a.
WBC		
**Median (×10^9^/L)**	14.4	n.a.
<100	259 (84)	n.a.
≥100	48 (16)	n.a.
PLT		
**Median (×10^9^/L)**	38	n.a.
>50	121 (39)	n.a.
≤ 50	186 (61)	n.a.
HGB		
**Median (g/L)**	77	n.a.
≥ 60	264 (86)	n.a.
<60	43 (14)	n.a.
Risk stratification		
Favorable	103 (34)	n.a.
Intermediate	132 (43)	n.a.
Adverse	72 (23)	n.a.
Response*		
CR	135 (77)	n.a.
No CR	41 (23)	n.a.

*Non-M3 patients evaluated after 2 cycles of treatment (n=176)

The bold values means statistically significant p-values.

n.a., not appliable.

### SNP selection and genotyping

Five immunosuppression-related genes were included: CIITA, MRC1, CD200, CD163 and LILRB4. Potentially functional SNPs were selected by using the NCBI dbSNP database and SNPinfo (https://snpinfo.niehs.nih.gov/). SNPs were chosen based on the following criteria (1): minor allele frequency (MAF) reported in HapMap was>5% for Chinese Han subjects (2); location in 5’ UTR and 3’ UTR, potentially affecting transcription activity or microRNA binding site capacity; and (3) low linkage disequilibrium with each other (R^2^<0.8). A total of nine SNPs were selected. The TaqMan genotyping for the SNP was performed on an ABI 7900 (Applied Biosystems, Foster City, CA, USA). All case/control status was carried out blind to the laboratory personnel. Genotyping of the proposed SNPs was all performed in the laboratory of Guangzhou.

### Statistical analysis

Genotype compliance with HWE among controls was assessed using a chi-square test. Differences in demographic characteristics between cases and controls were evaluated using chi-square tests. Age- and gender-adjusted ORs and 95% CIs for the relationships between SNPs and AML were determined by multivariate logistic regression analysis. Kaplan–Meier curves estimated OS, and Cox regression analysis evaluated prognostic factors of AML. All statistical analyses were performed using SPSS 26.0 software (SPSS Inc., Chicago, IL, USA). Statistical significance was defined as a two-tailed *p* value<0.05 or a false discovery rate (FDR) *q* value<0.05.

## Results

### SNP selection and study populations

The selected SNPs are listed in **Table 2**. Nine AML immunosuppression-related SNPs were selected, and eight were further analyzed after passing the HWE deviation and MAF>0.05 criteria. LILRB4 rs11540761 was excluded from subsequent analyses due to HWE deviations or unsuitability for the HapMap project. The characteristics of *de novo* AML patients and healthy controls are presented in **Table 1**.

**Table 2:** Selected genes and SNPs.

Gene	SNP	Variant	Variant allele	MAF	HWE (*p*-value)
**CIITA**	rs3087456	G>A	A	8.544303797%	0.882975526
	rs4780335	G>C	C	38.291139241%	0.825756208
**CD200**	rs2272022	C>A	A	11.234177215%	0.999977593
	rs3746444	A>G	G	13.449367089%	0.074491888
**CD163**	rs4883263	C>T	T	32.594936709%	0.503876686
**MRC1**	rs2253120	G>A	A	20.727848101%	0.888061888
	rs691005	T>C	C	35.126582278%	0.969425495
**LILRB4**	rs1048801	G>A	A	28.955696203%	0.468127859
	rs11540761*	G>T	T	12.816455696%	0.032893674

*SNPs was not included in further analysis.

### CD163 rs4883263 is associated with AML susceptibility

To assess the association between immunosuppression-related SNPs and AML susceptibility, preliminary screening with the chi-square or Fisher’s exact test was conducted on the control and AML groups under three genetic models. As shown in **Table 3**, CD163 rs4883263 was significantly correlated with the AML susceptibility under the co-dominant and dominant models (*p*<0.05). After adjusting for sex and age with FDR correction, the co-dominant CT genotype and dominant CT/TT genotype of rs4883263 were found to be protective factors against AML susceptibility compared to the CC genotype (*p*=0.014 and *p*=0.046, respectively).

**Table 3:** Association between SNPs and AML susceptibility.

Gene	SNP	Model	Genotype	Control (n)	AML case (n)	χ^2^ test *p* value	OR (95% CI)	Adjusted *p* value
CD163	rs4883263	Co-dominant	CC	139	160	**0.025**		
			CT	148	111		0.644 (0.454-0.914)	**0.014**
			TT	29	36		1.084 (0.619-1.899)	0.778
		Dominant	CC	139	160	**0.042**		
			CT+TT	177	147		1.397 (1.006-1.94)	**0.046**

SNP, single nucleotide polymorphisms; AML, acute myeloid leukemia; OR, odds ratio; CI, confidence interval.

The bold values means statistically significant p-values.

### Association of immunosuppression-related SNPs with the baseline data of AML patients

Among 307 AML patients, 255 were non-M3 AML. Considering the different treatment options and prognosis of M3 patients, we included only 255 non-M3 AML cases in the follow-up analysis. To further explore the value of immunosuppression-related SNPs in AML, the relationships between SNPs and baseline data at initial diagnosis were analyzed (**Table 4**).

**Table 4:** Association between SNPs and the baseline data of AML patients.

Gene	SNP	Model	Genotype	Normal chromosome karyotype	Abnormal chromosome karyotype	χ^2^ test *p* value	OR (95% CI)	Adjusted *p* value
CD163	rs4883263	Co-dominant	CC	86	52	**0.019**		
			CT	64	26		0.679 (0.382-1.204)	0.185
			TT	24	3		0.204 (0.058-0.713)	**0.013**
		Dominant	CC	86	52	**0.028**		
			CT+TT	88	29		0.548 (0.318-0.945)	**0.030**
		Recessive	CC+CT	150	21	**0.015**		
			TT	24	12		0.236 (0.069-0.811)	**0.022**
Gene	SNP	Model	Genotype	WBC< 100×10^9^/L	WBC≥ 100×10^9^/L	χ^2^ test *p* value	OR (95% CI)	Adjusted *p* value
CIITA	rs4780335	Co-dominant	GG	81	11	**0.046**		
			GC	103	24		1.969 (0.888-4.366)	0.095
			CC	25	11		2.978 (1.134-7.817)	**0.027**
		Recessive	GG+GC	184	35	**0.035**		
			CC	25	11		2.001 (0.884-4.530)	0.096
Gene	SNP	Model	Genotype	PLT> 50×10^9^/L	PLT≤ 50×10^9^/L	χ^2^ test *p* value	OR (95% CI)	Adjusted *p* value
CD163	rs4883263	Co-dominant	CC	58	80	**0.032**		
			CT	42	48		0.840 (0.49-1.439)	0.525
			TT	5	22		3.190 (1.132-8.992)	**0.028**
		Recessive	CC+CT	100	128	**0.011**		
			TT	5	22		3.422 (1.242-9.425)	**0.017**
CD200	rs2272022	Co-dominant	CC	74	124	**0.008**		
			CA	31	23		0.434 (0.234-0.804)	**0.008**
			AA	0	3		860325435.7 (0)	0.999
		Dominant	CC	74	124	**0.021**		
			CA+AA	31	26		0.488 (0.268-0.891)	**0.019**

SNP, single nucleotide polymorphisms; OR, odds ratio; CI, confidence interval.

The bold values means statistically significant p-values.

First, we analyzed the relationship between SNPs and BM blast percentage. A BM blast percentage of 70% or greater was considered hypercellular in the analysis. Initial screening using chi-square or Fisher’s exact test revealed no significant association between the selected SNPs and BM blasts (*p*>0.05).

Next, we analyzed the relationship between SNPs and chromosome karyotype. CD163 rs4883263 was related to abnormal chromosome karyotype in AML under three models (*p*<0.05). After adjusting for age and sex, the co-dominant TT genotype (OR=0.204, 95% CI=0.058-0.713, *p*=0.013), dominant CT/TT genotype (OR=0.548, 95% CI=0.318-0.945, *p*=0.030), and the recessive TT genotype (OR=0.236, 95% CI=0.069-0.811, *p*=0.022) of rs42883263 were identified as risk factors for AML abnormal karyotypes.

We further analyzed the relationship between SNPs and the levels of peripheral blood components in AML patients at initial diagnosis. The high WBC group included patients with WBC count ≥100×10^9^/L (19), and the low WBC group included those with WBC count <100×10^9^/L. The high PLT group had PLT levels >50×10^9^/L, and the low PLT group had PLT levels ≤50×10^9^/L. The high HGB group had HGB levels ≥60 g/L, and the low HGB group had HGB levels <60 g/L. As shown in **Table 4**, the chi-square test indicated that CIITA rs4780335 may be related to WBC count under the co-dominant and recessive models (*p*<0.05). After adjusting for age and sex, the CC genotype of CIITA rs4780335 under the co-dominant model was significantly associated with high WBC count at AML diagnosis (OR=2.978, 95% CI=1.134-7.817, *p*=0.027). For peripheral blood PLT count analysis, the chi-square test showed that CD163 rs883263 under co-dominant and recessive models, as well as CD200 rs2272022 under co-dominant and dominant models, were related to PLT count (*p*<0.05). After adjusting for age and sex, the TT genotype of CD163 rs883263 under the co-dominant model (OR=3.19, 95% CI=1.132-8.992, *p*=0.028) and recessive model (OR=3.422, 95% CI=1.242-9.425, *p*=0.017) was significantly associated with low PLT count at diagnosis. Additionally, the CA genotype of CD200 rs2272022 under the co-dominant model (OR=0.434, 95% CI=0.234-0.804, *p*=0.008), as well as the CA and AA genotypes under the dominant model (OR=0.488, 95% CI=0.268-0.891, *p*=0.019) were protect factors for low PLT count at diagnosis. No selected SNPs were associated with HGB content in the peripheral blood of AML patients (*p*>0.05).

### LILRB4 rs1048801 and AML treatment sensitivity

Of the non-M3 AML patients included in this study, 212 were treated, with 200 patients undergoing induction chemotherapy. After two cycles of treatment, 176 of 212 treated patients and 167 of 200 patients who underwent induction chemotherapy were evaluated for BM cytology. To elucidate the role of immunosuppression-associated SNPs in AML treatment response, we analyzed the relationship between SNPs and treatment response in 176 AML patients, and between SNPs and response to anthracycline/cytarabine chemotherapy in 167 AML patients. Our data showed a statistically significant relationship between SNPs and treatment response. As shown in **Tables 5** and **6**, LILRB4 rs1048801 was associated with non-remission (No CR) of BM morphology after 2 cycles of treatment under the co-dominant and dominant models (*p*<0.05). After adjusting for age and sex, the AG genotype of LILRB4 rs1048801 under co-dominant model (OR=0.349, 95% CI=0.163-0.750, *p*=0.007) and the AG/GG genotype under the dominant model (OR=0.352, 95% CI=0.167-0.743, *p*=0.006) were significantly associated with no CR status after 2 cycles of treatment (**Table 5**). Similarly, after adjusting for age and sex, the AG genotype of LILRB4 rs1048801 under the co-dominant model (OR=0.408, 95% CI=0.186-0.895, *p*=0.025) and the AG/GG genotype under the dominant model (OR=0.409, 95% CI=0.190-0.887, *p*=0.022) were significantly associated with no CR status after two cycles of anthracycline/cytarabine induction chemotherapy (**Table 6**). These results suggest that LILRB4 rs1048801 could significantly affect treatment sensitivity and that its effect on refractory AML patients is not strongly related to the type of chemotherapeutic agent.

**Table 5:** LILRB4 rs1048801 was associated with AML treatment response.

Gene	SNP	Model	Genotype	No CR	CR	χ^2^ test *p* value	OR (95% CI)	Adjusted *p* value
LILRB4	rs1048801	Co-dominant	AA	13	78	**0.013**		
			AG	25	50		0.349 (0.163-0.750)	**0.007**
			GG	3	7		0.374 (0.085-1.648)	0.193
		Dominant	AA	13	78	**0.003**		
			AG+GG	28	57		0.352 (0.167-0.743)	**0.006**

SNP, single nucleotide polymorphisms; No CR, non-remission; CR, complete remission; OR, odds ratio; CI, confidence interval.

The bold values means statistically significant p-values.

**Table 6:** LILRB4 rs1048801 was associated with AML induction chemotherapy response.

Gene	SNP	Model	Genotype	No CR	CR	χ^2^ test *p* value	OR (95% CI)	Adjusted *p* value
LILRB4	rs1048801	Co-dominant	AA	13	73	**0.034**		
			AG	23	48		0.408 (0.186-0.895)	**0.025**
			GG	3	7		0.412 (0.093-1.831)	0.244
		Dominant	AA	13	73	**0.001**		
			AG+GG	26	55		0.409 (0.190-0.877)	**0.022**

SNP, single nucleotide polymorphisms; No CR, non-remission; CR, complete remission; OR, odds ratio; CI, confidence interval.

The bold values means statistically significant p-values.

### ILIRB4 rs1048801 and CIITA rs4780335 are associated with AML OS

**Figure 1:**
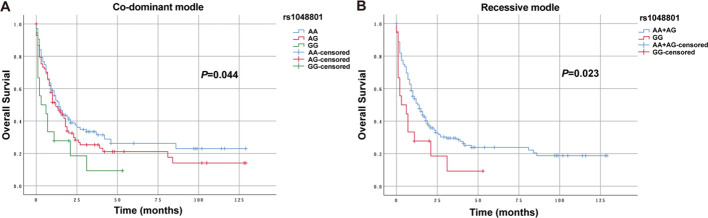
The overall survival of AML patients with AA, AG and GG genotypes in ILIRB4 rs1048801 under different models. **(A)** Co-dominant model. **(B)** Recessive model.

**Figure 2:**
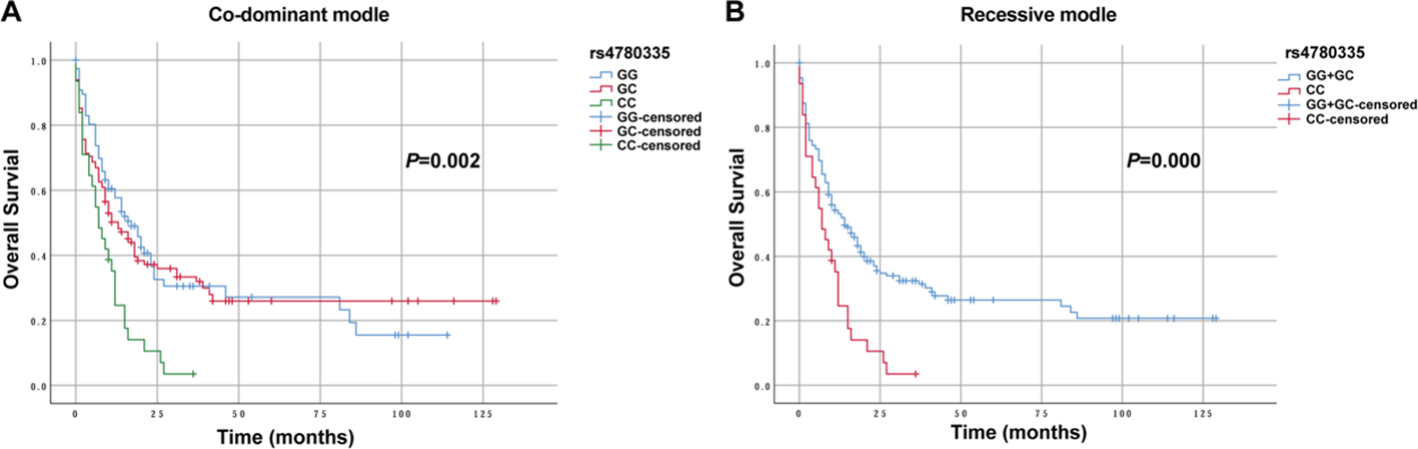
The overall survival of AML patients with GG, GC and CC genotypes in CIITA rs4780335 under different models. **(A)** Co-dominant model. **(B)** Recessive model.

Three genetic models were used to analyze the relationships between various SNPs and OS in non-M3 AML patients. Preliminary Kaplan–Meier screening revealed that the genotype frequency of rs1048801 in ILIRB4 and rs4780335 in CIITA was associated with prognosis under both co-dominant and recessive models (*p*<0.05) (**Figures 1**, **2**). Other SNPs showed no significant effect on OS. Under the co-dominant model of rs1048801 in ILIRB4, patients with the AA genotype (41 months) had better OS compared to those with the AA/AG genotypes (33 months and 12 months, respectively). Under the recessive model of rs1048801 in ILIRB4, patients with the GG genotype had significantly lower OS compared to those with the AA/AG genotypes. Under the co-dominant model of rs4780335 in CIITA, patients with the CC genotype (9 months) had lower OS compared to these with the GG (35 months) and GC genotypes (41 months). Similarly, under the recessive model of rs4780335 in CIITA, patients with the CC genotype had significantly lower OS compared to those with the GG/GC genotypes.

Patients aged 60 years or older had significantly shorter OS (9 months) than those younger than 60 years (43 months, *p*<0.001). Patients with a WBC count of 100×10^9^/L or more had a significantly lower OS (22 months) than those with a WBC count below 100×10^9^/L (40 months, *p*<0.001). Patients with HGB content less than 60 g/L had significantly lower OS (18months) than those with HGB content of 60 g/L or more (40 months, *p*<0.005). Patients with a PLT count below 50×10^9^/L had significantly lower OS (26 months) compared to those with 50×10^9^/L or more(51 months, *p*<0.005). Patients not receiving chemotherapy had significantly shorter OS (7 months) than those who received treatment (41 months, *p*<0.001). Additionally, OS was significantly lower in the patients with adverse risk stratification (22 months) compared to those with favorable and intermediate risks (76 months and 31 months, respectively; *p*<0.001).

### LILRB4 rs1048801 is associated with poor outcome in AML

**Figure 3:**
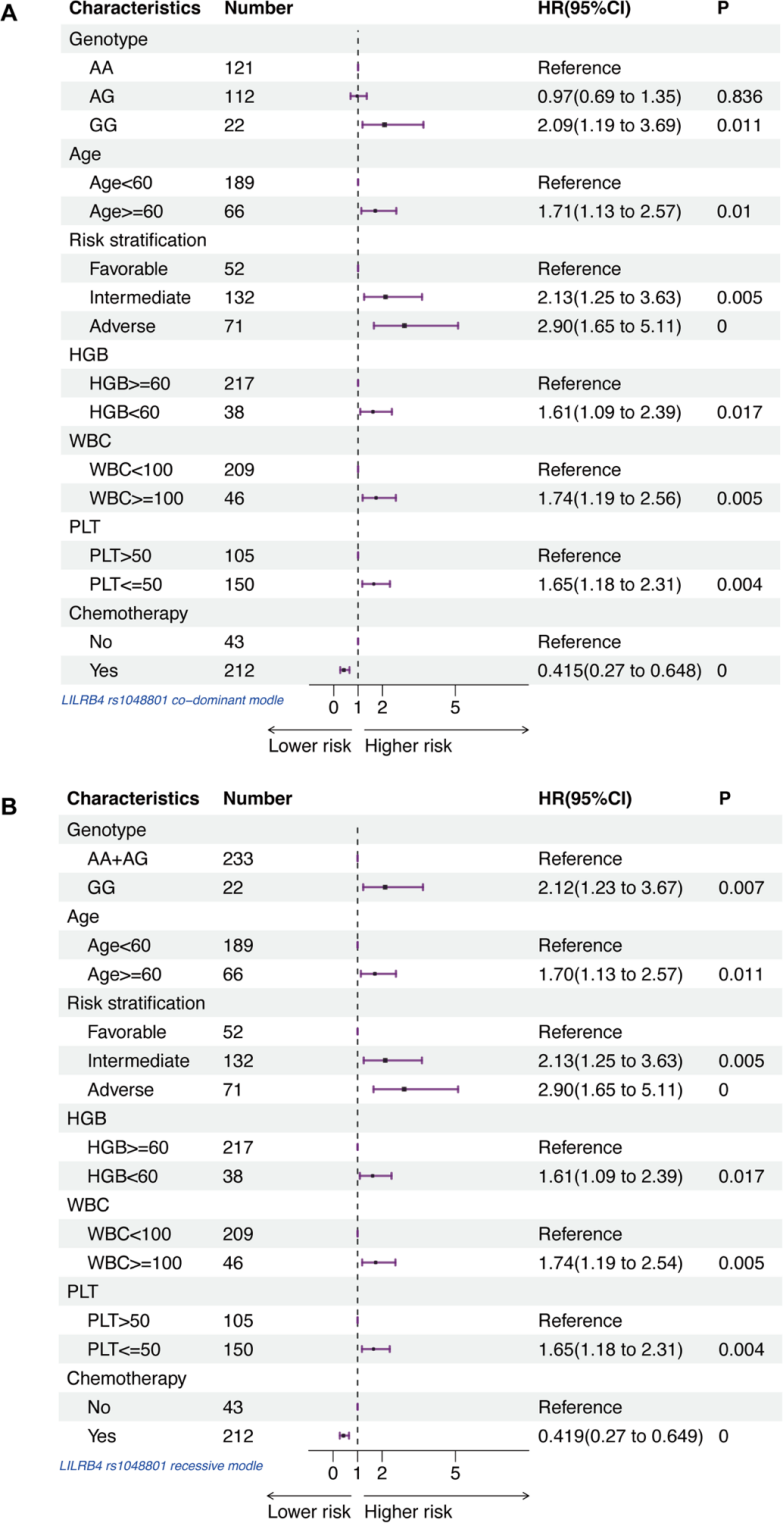
Forest plots of multivariable Cox proportional hazard models that includes AA, AG and GG genotypes in LILRB4 rs1048801, age, risk stratification, HGB content, WBC count, PLT count and chemotherapy reception for association with AML patients. **(A)** Co-dominant model. **(B)** Recessive model.

The presence of rs1048801 in LILRB4 or rs4780335 in CIITA, along with age, risk stratification, HGB, WBC and PLT count at diagnosis, and chemotherapy reception were included in a multivariate Cox regression analysis. After adjusting for age, risk stratification, HGB content, WBC count, PLT count and chemotherapy reception, the GG genotype of LILRB4 rs1048801 remained significantly associated with worse OS (**Figure 3**). The results indicated that the GG genotype of LILRB4 rs1048801 is an independent prognostic risk factor for AML under both the co-dominant (HR=2.09, 95% CI=1.19-3.69, *p*=0.011) and recessive models (HR=2.12, 95% CI=1.23-3.67, *p*=0.007). Conversely, the CIITA rs4780335 genotype is not an independent prognostic risk factor for AML.

### mRNA expression of CIITA and LILRB4 in AML patients

**Figure 4:**
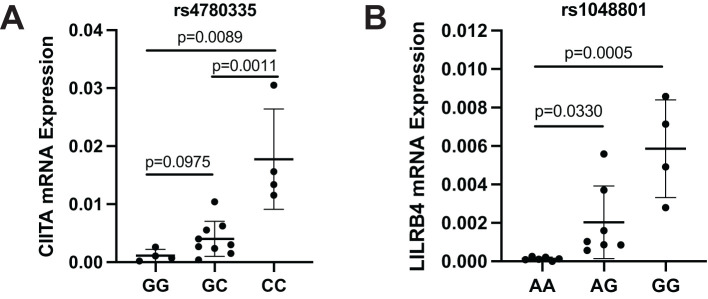
mRNA expression of CIITA and LILRB4 in AML patients with different genotypes of rs4780335 and rs1048801. **(A)** Expression of CIITA mRNA in AML patients with the GG, GC and CC genotype (n = 4, n = 9 and n = 4, respectively). **(B)** Expression of LILRB4 mRNA in AML patients with the AA, AG and GG genotypes (n = 6, n = 7 and n = 4, respectively).

To further analyze the potential functional consequences of key SNPs that may affect overall survival, we evaluated the effects of CIITA rs4780335 and ILIRB4 rs1048801 polymorphisms on mRNA expression using AML patients. CIITA rs4780335 showed significantly increased mRNA expression in patients carrying the CC genotype compared to those carrying the GG and GC genotypes (**Figure 4A**), and ILIRB4 rs1048801 also showed increased mRNA expression in patients carrying the GG and AG genotypes compared with those carrying the AA genotype (**Figure 4B**). In summary, the results showed that CIITA rs4780335 and ILIRB4 rs1048801 mutations may affect the expression levels of their own mRNAs, respectively, and are associated with the prognosis of AML.

### CD163, CIITA, LILRB4 and CD200 are highly expressed in AML patients

**Figure 5:**
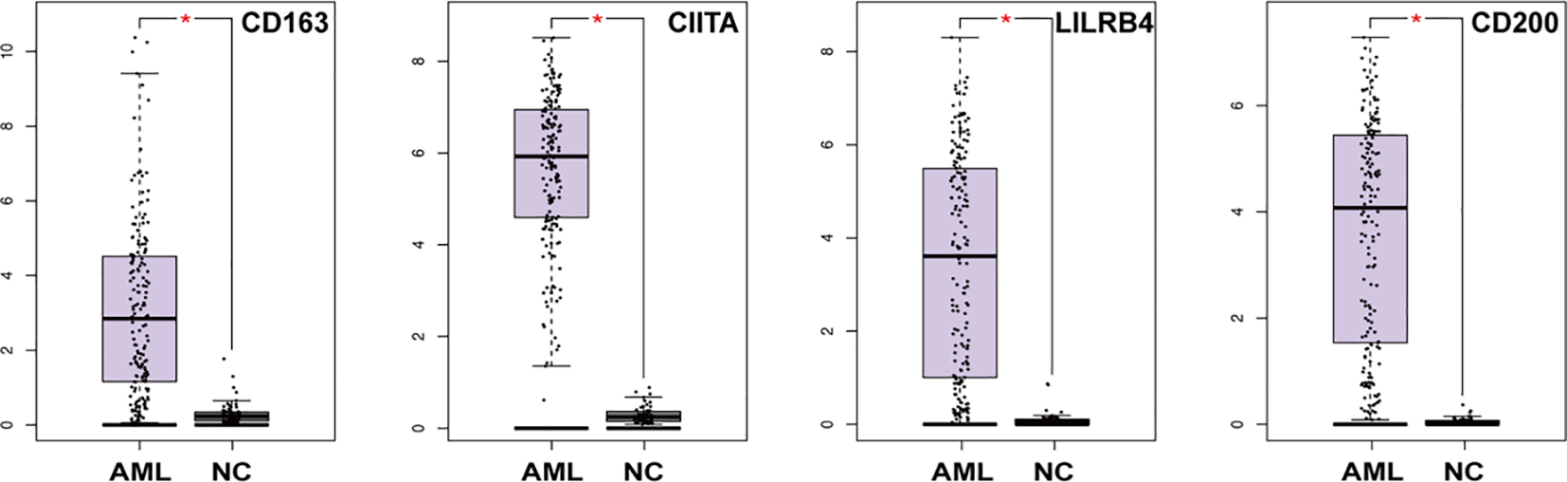
mRNA expression of CD163, CIITA, LILRB4 and CD200 in AML patients and normal control in the TCGA dataset (173 in the AML group and 70 in the NC group). *P < 0.05.

Further, the expression differences of CD163, CIITA, LILRB4, and CD200 in AML patients and normal controls were retrieved by Gene Expression Profiling Interactive Analysis (GEPIA). As shown in **Figure 5**, we found that the expression of these molecules in AML in the TCGA database was higher than in the control group (*p*<0.05). This suggests that rs4883263 in CD163, rs4780335 in CIITA, rs1048801 in LILRB4 and rs2272022 in CD200 may play a role in AML by affecting gene expression.

## Discussion

**Figure 6:**
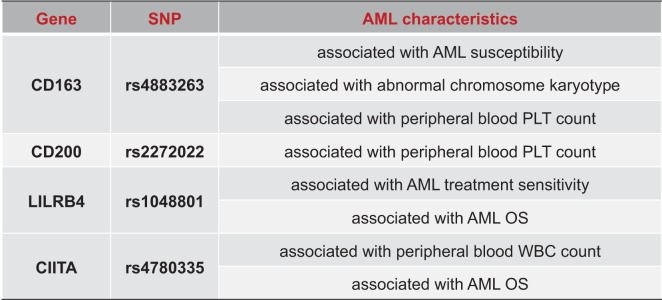
Summary of associations between SNP and AML characteristics.

A growing body of research has highlighted immune gene polymorphisms and their involvement in the pathogenesis and progression of AML. Earlier studies mostly centered on SNPs affecting immune checkpoint molecules and cytokines, whereas this study innovatively focused on five newly reported AML immune suppression-related genes identified by scRNA-Seq and RNA-Seq, and further explored the association between SNPs within these genes and AML. Comprehensive statistical analysis showed that rs4883263 in the CD163 gene was associated with AML susceptibility, chromosomal karyotype abnormalities, and low PLT count at diagnosis. Conversely, rs2272022 in CD200 was a protective factor for low PLT count. rs4780335 in CIITA was associated with high WBC count at diagnosis and worse OS. Additionally, rs1048801 in LILRB4 was associated with worse AML treatment response, lower OS, and may be an independent prognostic risk factor (**Figure 6**). We also found that the expression of CD163, CIITA, LILRB4, and CD200 was higher in AML patients than in normal controls. These results suggest that immunosuppression-related genes are involved in AML pathogenesis and development. Our findings may serve as potential therapeutic response indicators for guiding clinical treatment of AML.

The impact of genetic polymorphisms on the efficacy of induction chemotherapy and targeted therapy in AML patients has been widely studied. LILRB4 is expressed at higher levels in primary human AML cells than in normal cells (20, 21). Activation of LILRB4 in AML cells can inhibit T cell proliferation and promote AML cell migration and infiltration (12, 22). Dobrowolska et al. demonstrated that LILRB4 was co-expressed with the leukemic stem cell (LSC) markers CD34 and CD117 in 39% and 50% of cases, respectively (21), suggesting that LILRB4 is a highly sensitive and specific marker important for differential diagnosis. Chimeric antigen receptor (CAR-T) cells, antibody-drug conjugates (ADCs) targeting LILRB4, and biomimetic inhibitors are actively under investigation (23, 24). However, no study has reported a correlation between LILRB4 SNPs and AML. Here, we showed that LILRB4 rs1048801 was associated with poor AML treatment response after two cycles treatment (either induction chemotherapy or targeted therapy), significantly affecting treatment sensitivity regardless of the type of chemotherapeutic drugs. Under the co-dominant and recessive models, patients with the GG genotype of LILRB4 rs1048801 had significantly lower OS. Moreover, it may be an independent prognostic risk factor for AML after multivariate Cox regression analysis. This is the first report that LILRB4 SNPs are associated with AML, which is significant for using LILRB4 rs1048801 to evaluate the efficacy and prognosis of AML. However, the results need further confirmation in studies with larger sample sizes.

Additionally, we found that CIITA rs4780335 was associated with high WBC count at diagnosis and was related to worse OS. CIITA is a master regulator of MHC class II (MHC II) expression. Studies have shown that CIITA expression levels are significantly higher in AML cases with a first remission duration of less than two years (10). In AML, relapse after allogeneic transplantation is associated with loss of major MHC II expression (25–27). Reduced expression of the MHC II transcriptional coactivator CIITA has been observed in some AML cases (25), but MHC II expression has also been lost despite unchanged or increased CIITA expression (26), suggesting additional mechanisms of immune escape. CIITA SNPs have previously been shown to be associated with susceptibility to several immune mediated disorders and chronic hepatitis B virus infection (28, 29), whereas their association with AML has not been reported. In this study, the CC genotype of CIITA rs4780335 under the co-dominant model was significantly associated with a high WBC count at AML diagnosis. For OS analysis, under the co-dominant and the recessive models of rs4780335 in CIITA, patients with the CC genotype had significantly worse OS compared to those with the GG and GC genotypes. These results demonstrate that CIITA SNPs may be important in assessing the prognosis of AML.

CD163 rs4883263 was newly found to be associated with AML susceptibility, chromosomal karyotype abnormalities, and low PLT count at diagnosis in our study. CD163 is considered a potential therapeutic target for macrophage-directed therapy in cancers such as glioma and gastric cancer (30). In AML, high expression of CD163 is associated with poor OS and is significantly correlated with AML prognosis, providing a basis for developing targeted drugs for AML with high CD163 expression (31, 32). Researchers found that CD163 SNPs exhibited significant correlations with classical Hodgkin’s lymphoma (CHL) and may be a predictive biomarkers for CHL prognosis (33), suggesting an important role for CD163 SNPs in predicting the prognosis of hematological tumors. These studies are consistent with our findings on the association of CD163 SNP with AML susceptibility. Moreover, CD163 rs4883263 was associated with abnormal chromosome karyotype and low peripheral blood PLT count at diagnosis, suggesting a potential link between the SNP and poor prognosis of AML. In addition, CD200 rs2272022 seemed to be a protective factor for low peripheral blood PLT count. CD200 belongs to the immunoglobulin superfamily and acts as an immunosuppressive signal through the receptor CD200R on immune cells. In AML, CD200 is considered a novel LSC marker, which is highly expressed and associated with poor OS (34, 35). As a protective factor against low platelet counts, the potential mechanism of CD200 rs2272022 in AML deserves further exploration.

Based on multi-omics research progress, this study selected immunosuppression-related genes expressed by AML cells. The innovative combination of single-cell screening targets and SNP analysis was used to study the correlation between immunosuppression-related SNPs and AML pathogenesis and treatment response, greatly improving the reliability of the results and becoming the biggest advantage of this study. Given that SNPs are inherited mutations that can be efficiently assessed irrespective of cell type, and that genotyping assays are both widely accessible and capable of rapid turnaround, preemptive genotyping can be readily conducted in most clinical settings using various sample types, including blood, buccal swabs, or skin. Consequently, the prospective investigation of these germline polymorphisms in clinical laboratories is highly feasible. Our findings offer an opportunity to further refine personalized immunotherapy regimens through genomic profiling of patients, by expanding the study cohort to elucidate the prognostic significance of LILRB4 rs1048801 and CIITA rs4780335.

However, the exact molecular and cellular mechanisms of immunosuppression for the identified SNPs require further investigation. In future studies, we will use CRISPR editing and other technologies to functionally validate key SNPs and clarify the mechanisms by which these SNPs affect the pathogenesis and development of AML. Furthermore, there are limitations to the analysis between SNPs and AML in this study. First, due to the presence of confounding factors such as no uniform chemotherapy and other comorbidities, the statistical model used in this study has certain limitations. In addition, the limited size of both AML cases and controls may have constrained the robustness and comprehensiveness of the multivariate analysis across all variables. Moreover, given the heterogeneity of the AML patient population and the limited sample size in this single-center study, these findings require validation through larger, multi-center cohort studies.

## Conclusions

Recent multi-omics sequencing data showed that five immunosuppression-related genes, CIITA, CD200, CD163, MRC1, and LILRB4, are associated with the progression of AML, but their SNP associations have not been reported. Our study found that these immunosuppression-related SNPs are indeed related to the different occurrence, development, and treatment processes of AML, such as CD163 rs4883263 is associated with AML susceptibility, abnormal chromosome karyotype, and low PLT count at diagnosis; CD200 rs2272022 is a protective factor against low PLT count; CIITA rs4780335 is associated with high WBC count at diagnosis and poor OS; LILRB4 rs1048801 is associated with poor AML treatment response and poor OS, respectively. In particular, LILRB4 rs1048801 can be used as an independent prognostic factor for AML, suggesting that immunosuppression-related SNPs are closely related to the progression and treatment of AML and should be taken seriously.

## Data Availability

The datasets presented in this study can be found in onlinerepositories. The names of the repository/repositories and accessionnumber(s) can be found in the article/**Supplementary Material**.
